# Metalloproteinases and Their Inhibitors: Potential for the Development of New Therapeutics

**DOI:** 10.3390/cells9051313

**Published:** 2020-05-25

**Authors:** Maryam Raeeszadeh-Sarmazdeh, Linh D. Do, Brianne G. Hritz

**Affiliations:** Chemical and Materials Engineering Department, University of Nevada, Reno, NV 89557, USA; linhd@nevada.unr.edu (L.D.D.); bhritz@nevada.unr.edu (B.G.H.)

**Keywords:** metalloproteinases, metzincins, matrix metalloproteinases, MMPs, a disintegrin and metalloproteases, ADAMs, tissue inhibitors of metalloproteinases, TIMPs, MMP inhibitors, MMP-responsive therapeutics

## Abstract

The metalloproteinase (MP) family of zinc-dependent proteases, including matrix metalloproteinases (MMPs), a disintegrin and metalloproteases (ADAMs), and a disintegrin and metalloproteinase with thrombospondin motifs (ADAMTSs) plays a crucial role in the extracellular matrix (ECM) remodeling and degradation activities. A wide range of substrates of the MP family includes ECM components, chemokines, cell receptors, and growth factors. Metalloproteinases activities are tightly regulated by proteolytic activation and inhibition via their natural inhibitors, tissue inhibitors of metalloproteinases (TIMPs), and the imbalance of the activation and inhibition is responsible in progression or inhibition of several diseases, e.g., cancer, neurological disorders, and cardiovascular diseases. We provide an overview of the structure, function, and the multifaceted role of MMPs, ADAMs, and TIMPs in several diseases via their cellular functions such as proteolysis of other cell signaling factors, degradation and remodeling of the ECM, and other essential protease-independent interactions in the ECM. The significance of MP inhibitors targeting specific MMP or ADAMs with high selectivity is also discussed. Recent advances and techniques used in developing novel MP inhibitors and MP responsive drug delivery tools are also reviewed.

## 1. Introduction

Metzincins consist of a large heterogeneous superfamily of zinc-dependent endopeptidases present in the extracellular matrix (ECM). The metzincin family of metalloproteinase (MP) includes matrix metalloproteinase (MMP), ADAM (a-disintegrin and metalloproteinase), and ADAMTS (a-disintegrin and metalloproteinase with thrombospondin motifs) [1]. The metalloproteinases (which we refer to MMPs, ADAMs, and ADAMTSs) play a critical role in remodeling of the ECM by proteolytic degradation of ECM components, activation of cell surface proteins, and shedding of membrane-bound receptor molecules. They regulate activity of other proteinases, growth factors, chemokines, and cell receptors, and mediate several biological activities such as cell migration, differentiation, proliferation, and survival [2] in various forms of cellular function. There are 23 different members of MMPs, 21 of ADAMs, and 19 of ADAMTSs known to date in humans [3].

**Figure 1:**
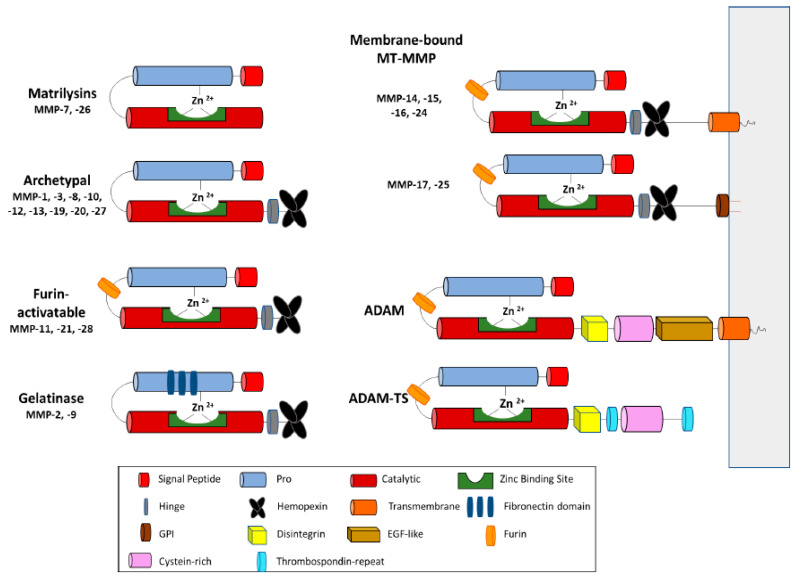
Schematic representation of matrix metalloproteinases (MMPs), a-disintegrin and metalloproteinases (ADAMs), and a-disintegrin and metalloproteinase with thrombospondin motifs (ADAMTSs). The structural domains of different metalloproteinases (MPs) are displayed. GPI, Glycosylphosphatidylinositol-anchoring sequence; EGF, epidermal growth factor-like domain.

These proteases are classified based on various criteria, such as their substrate preferences, mechanism of enzymatic reaction, soluble or transmembrane domains, and structural homology. The major structural homology which was found in all proteins of this superfamily is highly conservative motif HEXXHXXGXXH present within the active site of the enzyme [4]. The majority of differences between zinc-dependent metalloproteases are associated with the occurrence of additional domains within the C-terminus of these proteins [5] (Figure 1).

Metalloproteinase (MP) activity is tightly regulated by proteolytic activation of the zymogen form and its natural inhibitor, tissue inhibitor of metalloproteinases (TIMPs). Under pathologic conditions, overexpression of metalloproteinases or insufficient control of TIMPs results in the dysregulation of tissue remodeling, causing a variety of diseases such as cancer [6,7], neurodegenerative disease [8,9], arthritis, cardiovascular diseases [10,11], and fibrotic disorders [12,13]. Although early efforts of targeting MMPs largely failed in later stages of clinical trials, metalloproteinases remain a highly desirable therapeutic target based on their key role in progression of several diseases [6].

Different classes of MP inhibitors were developed and tested including small molecules, peptides, and protein-based binders such as antibodies and TIMPs. With recent advances in protein engineering and design, from recruiting better understanding of the structure and function of these metalloproteinases to state-of-the-art techniques such as directed evolution and high throughput screening, new classes of therapeutics targeting MMPs with high affinity and selectivity are on the rise [6]. Design of “smart,” MMP-responsive therapeutics and drug delivery vehicles also enhanced site-specific drug delivery to tumor sites, where MMPs are upregulated [14]. Among all MPs, the role of MMPs and their inhibitors were studied more extensively [15]; however, we also included the role of ADAMs and ADAMTSs in developing several diseases in this review. This review focuses on therapeutic applications for metalloproteases as targets for inhibition and as tools for drug activation. It has the following sections:

- MP structure and function in ECM
- MPs in cell signaling
- MPs in cancer
- MPs in central nervous system and neurodegenerative diseases
- MPs in cardiovascular diseases
- MPs in fibrosis and other diseases
- MMP inhibition for developing therapeutics
- MMP-responsive drugs and drug delivery tools
- Conclusion and future directions

## 2. MP Structure and Function in ECM

The structure of MPs contains a propeptide sequence and a catalytic domain. MMP structure also includes a hinge region and a hemopexin (PEX) domain [4,16]. Based on their structural domains, MMPs have been classified into collagenase, gelatinase, stromelysin, matrilysin, and membrane-bound MMPs (MT-MMPs) [6,17] (Figure 1). MT-MMPs contain a transmembrane or Glycosylphosphatidylinosotol (GPI)-anchored domain at their C-terminus. MT-MMPs are anchored to the cell membrane via a covalent bond. The secreted MMPs can localize to the cell surface by binding interactions to cell-surface associated proteins such as CD44. Other binding interactions include heparan sulfate proteoglycans, collagen type IV, or extracellular matrix metalloproteinase inducer (EMMPRIN) [18]. Both soluble and MT-MMPs are essential for diverse physiological pathological processes that are involved with both extracellular matrix remodeling and pericellular proteolysis [19].

ADAMs are membrane-anchored metalloproteinases. They have similar catalytic domains to MMPs; however, they do not have a PEX domain, and instead possess three additional epidermal growth factor (EGF)-like domains along with the disinterring domain. ADAMTS family members contain a variable number of type-1 thrombospondin (TSP-1) domains at the C-terminal region [3] (Figure 1).

The propeptide domain of MMPs is highly conserved, and it is the switch sequence that interacts with Zn^2+^. The cystine within this region is what allows the MMP to either be in the active or inactive state [20]. The catalytic domain is characterized as being the zinc-binding motif which, in the active state, will disassociate from the propeptide domain [16]. Hinge regions are mainly used to allow movement between the catalytic and hemopexin domain. MMPs are synthesized as inactive zymogens. In the zymogen (pro-MMP) form, the pro-domain interacts through the sulfhydryl group of the cysteine with the catalytic zinc ion and inhibits MMP proteolytic activities. MMP activation requires proteolytic removal of the pro-domain, usually by other MMPs or serine proteases outside the cell with the exception of MMP-11 and MMP-28, as well as some of the MT-MMPs, which are activated by intracellular furin-like serine proteinases before reaching the cell surface [6].

MMPs with gelatinase activity hydrolyze gelatin into polypeptides, peptides, and amino acids that can then be secreted through the cellular membrane. MMP-2 and MMP-9 belong to gelatinases and facilitate both gelatin and collagen-binding through three fibronectin type-II-like repeat domains inserted in the catalytic domain of the structure [21]. MMP-9 also contributes to wound healing suppression, where overexpression of MMP-9 results in a leakage in the healing process attributing to the continuous break down of collagen [22]. The family of collagenases, consisting of MMP-1, MMP-8, and MMP-13, cleave collagen, resulting in breakdown of joint cartilage, which contributes to inflammation [23]. The MMP members of the stromelysin family, including MMP-3 and MMP-10, directly degrade non-collagen connective tissues, including but not limited to proteoglycans, fibronectin, and laminin, while also interacting with other MMPs to perform other degradation mechanisms [24]. The matrilysin class of MMPs, including MMP-7, is found to directly cleave elastin, type II collagen, fibronectin, vitronectin, aggrecan, and proteoglycan [25].

Similar to MMPs, ADAMs carry proteolytic function by degrading ECM components and regulate intermolecular interactions by interacting with cell receptors and cell signaling components. On the other hand, ADAMTS proteases have a conserved role in the human organism as proteases, with some differentiation in terms of substrate specificity [26].

## 3. MMP Regulation in the ECM

Metalloproteinase activity is tightly regulated by activation of inactive zymogens and natural inhibition by endogenous inhibitors. Zymogen activation occurs by proteolytic cleavage of the pro-domain protecting the catalytic site. Several MMPs participate in activation of other pro-MMPs, usually leading to a cascade of activations. For example, MMP-3 and MMP-10 activate MMP-1, MMP-7, MMP-8, and MMP-9, enhancing ECM degradation [21], whereas MMP-14 activates both MMP-2 and MMP-13 in the presence of TIMP-2. MMP-2 and MMP-13 were shown to aid the cleavage of the pro domain in pro-MMP-9 [24], which was shown to affect tumor invasion and metastasis by promoting cell migration [22]. This complex network of MMP cross-activation is able to destruct the ECM if the tight regulation of MMPs is compromised [26].

Tissue inhibitors of metalloproteinases (TIMPs), a family of four members in humans, are endogenous inhibitors of MPs and regulators of MMPs and ADAMs’s function, forming a tight 1:1 stoichiometric inhibitory complex [27]. TIMPs consist of two domains that are packed side-by-side, each domain is stabilized by three internal disulfide bonds [28,29]. The N-terminal domain of TIMPs is known as the “inhibitory domain” since the isolated N-terminal domain of TIMPs were found to independently inhibit MMP. The conserved core epitope centered around the N-terminal strand, including the ‘Cys-X-Cys’ motif, is the major interaction site of TIMP with an MMP catalytic domain, which coordinates to the active-site divalent catalytic zinc ion of the MMP, thus blocking the substrate-binding cleft [30]. However, full-length TIMP-1 was shown to provide stronger MMP inhibition [31]. The cooperation between two domains of TIMP-1 in improving inhibition of MMP-3 is another indication for the importance of the TIMP-1 C-terminal domain in TIMP/MMP interactions and inhibition of MMPs [32].

TIMPs have broad, overlapping specificity in binding and inhibition of MMPs, ADAMs, and ADAMTSs ranging from 0.6 fM for the association of TIMP-2 with MMP-2 [33], to the high nanomolar range for the relatively poor inhibition of MMP-14 and MMP-15 by TIMP-1. The non-inhibitory interactions can also occur between select TIMPs and MMPs. TIMP-1 and TIMP-2 interact with the PEX domains of MMP-2 and MMP-9, resulting in some of the strongest interaction of TIMP/MMP pairs [34,35,36]. The C-terminal domains of TIMP-2, TIMP-3, or TIMP-4 can bind to the hemopexin domain of MMP-2 or pro-MMP-2, while the C-terminal domains of TIMP-1 or TIMP-3 can associate with MMP-9 or pro-MMP-9 [37]. TIMP-2 uniquely interacts with pro-MMP-2 [38], which is shown to be important in MMP-2 activation by MMP-14 [39]. The TIMP-1 C-terminal domain is also shown to interact with pro-MMP-9 hemopexin domain and the protect the secreted proenzyme from activation by other MMPs both in vitro and in vivo [30,36,40,41,42,43]. TIMPs also inhibit some of the ADAM and ADAMTS family of MP through similar interactions with MMPs. Among all four TIMPs, TIMP-3 was found to inhibit ADAM-17 and ADAM-12 [44,45], as well as ADAMTS-1, ADAMTS-4, and ADAMTS-5 [46,47], both TIMP-1 and TIMP-3 could inhibit ADAM-10 [44].

Dysregulation of MMPs can affect many physiological processes such as morphogenesis, angiogenesis, tissue remodeling (e.g., cardiovascular remodeling), embryonic development, regulation of cell growth and death, and wound healing [11,48]. In most typical adult tissues, MMPs remain at a rather low expression level, and a balance between MMPs and Tissue Inhibitors of Metalloproteinases (TIMPs) maintain the ECM equilibrium [37]. However, with inflammation and certain demand in remodeling activities, MMP expression increases causing an imbalance of MMP and TIMP levels [48,49].

MMP expression and activity is strictly controlled by several metabolic pathways and cell signaling molecules. Tumor necrosis factor (TNF)-α was shown to upregulate the collagenase class of MMPs and downregulate TIMP-1 expression [50]. The ERK ½ pathway is also known to impact transcription of several MMPs and when the ERK ½ pathway is inhibited, MMP transcription is downregulated [51]. KISS1R, a G protein coupled receptor (GPCR), was identified to regulate the phosphorylation of MT1-MMP and interacts with the ERK ½ pathway which is known to play a role in several stages of cancer [52]. Hypoxia, lack of oxygen in lungs caused by Mycobacterium tuberculosis, was also shown to upregulate the transcription of MMP-1 due to the lack of regulation that dimethyl oxalyl glycerin has on the hypoxia-inducible factor (HIF)-1 [53]. Relaxin-2, a peptide hormone, has been attributed to greater anterior cruciate ligament (ACL) injuries in females due to its role in upregulation of MMP transcription in the MMP pathway and downregulation of the expression of TIMP-1 [24]. 

RD1 genes, encoding early secretory antigenic target (ESAT-6), were also found to be involved in the upregulation of MMP-10, which interacts with MMP-1 causing higher collagenase activity in patients diagnosed with tuberculosis [54]. Along with several signaling pathways that regulate various MMPs, there are also various transcription factors such as nuclear factor kB (NF-kB) and signal transducer and activator of transcription-3 (STAT-3), that transcribe various MMPs with a role in the metastasis in cancer [55]. NF-kB contains regions of DNA-binding and dimerization domains, nuclear translocation signal (NLS), and binding site for the inhibitor of kappa B (IkB). In spontaneously hypertensive rate (SHR), higher levels of NF-kB were found, which are responsible for inducing MMP expression [56]. It was also identified that NF-kB is a key factor in macrophage-derived MMP-1 and MMP-3 secretion; however, if the NF-kB pathway is inhibited, the MMP expression is negatively affected. This finding clarified the role of NF-kB inhibition through drug treatment, and it was also identified that the NF-kB knockout leads to severe phenotypes or lethality [57]. It has also been identified that when NF-kB affects MMP-9 transcription; MMP-9 transcription is improved by two-fold when NF-kB is present [58]. Overall, although the inhibition of NF-kB is known to reduce transcription of various MMPs, it usually did completely knockdown the MMPs expression.

EGFR, integrin-β1, integrin-α2, myosin regulatory light chain (MRLC), and YAP (yes-associated protein) were also found to enhance MMP-7 expression through the generation of a positive feedback loop. Upregulation of integrin-β1, integrin-α2, and EGFR phosphorylation led to MRLC phosphorylation, causing YAP activation which in turn led to enhancement of MMP-7 expression [25]. Syndecan-2, a heparan sulfate proteoglycan, is located on the cell surface, attributes to cell adhesion, and activates membrane localization of PKC-γ. Activating PKC-γ will then cause activation of FAC/ERK signaling and therefore result in upregulation of MMP-7 [59].

## 4. MPs and Their Inhibitors in Cell Signaling

Matrix Metalloproteinases (MMPs) have been identified to contain a central role in various types of cells. MMPs are well-known for degrading structural components of the ECM. However, cleavage of ECM proteins may trigger cellular receptors for structural ECM components that affects cell signaling and behavior [18]. MMPs and ADAMs also accept substrates other than ECM components such as growth-factor receptors, cell-adhesion molecules, and chemokines. The cross-reactivity and interactions of the metalloproteinase network as well as overlapping substrate recognition make it difficult to understand the individual role of each MMP in the ECM.

Beyond the degradation of ECM components, several types of MMPs and ADAMs are known for proteolysis of growth factors such as epidermal growth-factor (EGFR), HER2/neu (or ERBB2), HER4 (or ERBB4), and the hepatocyte-growth-factor receptor (HGFR) in the c-MET signaling pathway [49]. For instance, MMP-2 plays a role in cleavage of fibroblast growth factor receptor-1 (FGF). MMPs are also known to target chemokines which affect infiltration and migration of leukocytes. MMP-9 is known to cleave CXCL8 (also known as IL-8) as a substrate [49]. Thus, MPs are known in regulating of inflammatory and immune responses. 

MMPs are also known to interact with ADAMs which are capable of degrading aggrecan, a cartilage-specific proteoglycan core protein [60]. MMPs and ADAMs are both involved with the c-MET signaling pathway that involves the binding of HGF, causing tyrosine residues Y1234 and Y1235 to phosphorylate, which in turn allows them to recruit signaling effectors with important functions in cell viability and motility [61]. ADAMs, such as ADAM-10 and ADAM-17, are also involved with cell-cell communication through the Notch signaling pathway and help to determine cell fate during development [62]. The ADAM cleavage site of Notch receptor in a protease-resistant state is protected by a negative regulatory region (NRR). The ligand-bearing cells induce global conformational changes in Notch that unfold the NRR structure and expose the ADAM cleavage site, initiating proteolytic activation of the Notch receptor. Both ADAM10 and ADAM17 were found to accept Notch1 (N1) as a substrate, however, the particular ADAM required for receptor activation is dependent on signaling induced by ligands [63]. Overexpression of ADAM will lead to a higher abundance of Notch cleavages which can result in the production of cancerous cells. MMPs and ADAMs interact with many other substrates that can cause a pyramid effect in cellular signaling [63,64]. 

Tissue inhibitors of metalloproteinases (TIMPs) also hold an important role in cell signaling as they inhibit MMPs and ADAMs which restrict the formation of cancer, inflammatory, and degenerative diseases. TIMPs are necessary to maintain cellular hemostasis since many MMPs interact with varying substrates, which necessitates the four groups of TIMPs for higher-affinity binding to the various MMPs and ADAMs [27]. Non-selective inhibition of MMPs could also be detrimental to the cell since MMPs are regulators for the ECM and the degradation of collagen is necessary for adequate survival [65]. In retrospect, if the opposite occurs and TIMP is unable to inhibit MMPs efficiently, or if there is overexpression of the mRNA of MMPs, the excessive degradation of the extracellular membrane leads to inconsistent regulation and initiation and progression of cancer or various diseases. Research recently has been leading towards either identifying chemicals or different pathways that can inhibit MMPs without adding TIMPs, such as hispidulin which induces TIMP expression [66]. Overall, a perfect balance between the various MMPs and TIMPs are necessary for proper hemostasis of every cell to limit the creation of various cancers and diseases. MMPs are also known to regulate angiogenesis, a critical step in tumor growth. MMP-2, -9, and -14 directly regulate angiogenesis [67,68,69]. 

MMPs are also known to interact with ADAMs, which are capable of degrading aggrecan, a cartilage-specific proteoglycan core protein [61]. MMPs and ADAMs are both involved with the c-MET signaling pathway that involves the binding of HGF, causing tyrosine residues Y1234 and Y1235 to phosphorylate, which in turn allows them to recruit signaling effectors with important functions in cell viability and motility [62]. ADAMs, such as ADAM-10 and ADAM-17, are also involved with cell–cell communication through the Notch signaling pathway and help to determine cell fate during development [63]. The ADAM cleavage site of Notch receptor in a protease-resistant state is protected by a negative regulatory region (NRR). The ligand-bearing cells induce global conformational changes in Notch that unfold the NRR structure and expose the ADAM cleavage site, initiating proteolytic activation of the Notch receptor. Both ADAM10 and ADAM17 were found to accept Notch1 (N1) as a substrate; however, the particular ADAM required for receptor activation is dependent on signaling induced by ligands [64]. Overexpression of ADAM will lead to a higher abundance of Notch cleavages, which can result in the production of cancerous cells. MMPs and ADAMs interact with many other substrates that can cause a pyramid effect in cellular signaling [64,65].

Tissue inhibitors of metalloproteinases (TIMPs) also hold an important role in cell signaling as they inhibit MMPs and ADAMs, which restrict the formation of cancer, inflammatory, and degenerative diseases. TIMPs are necessary to maintain cellular hemostasis since many MMPs interact with varying substrates, which necessitates the four groups of TIMPs for higher-affinity binding to the various MMPs and ADAMs [27]. Non-selective inhibition of MMPs could also be detrimental to the cell since MMPs are regulators for the ECM and the degradation of collagen is necessary for adequate survival [66]. In retrospect, if the opposite occurs and TIMP is unable to inhibit MMPs efficiently, or if there is overexpression of the mRNA of MMPs, the excessive degradation of the extracellular membrane leads to inconsistent regulation and initiation and progression of cancer or various diseases. Research recently has been leading towards either identifying chemicals or different pathways that can inhibit MMPs without adding TIMPs, such as hispidulin which induces TIMP expression [67]. Overall, a perfect balance between the various MMPs and TIMPs are necessary for proper hemostasis of every cell to limit the creation of various cancers and diseases. MMPs are also known to regulate angiogenesis, a critical step in tumor growth. MMP-2, -9, and -14 directly regulate angiogenesis [68,69,70].

## 5. MPs in Cancer

MPs have long been held responsible for cancer cell invasion and metastasis because of the great impact of metalloproteinases have in remodeling the ECM of tumors. MPs and their proteolytic function are responsible in several aspects of cancer such as cancer-cell growth, differentiation, apoptosis, migration, invasion, and tumor angiogenesis [49]. They have significant roles in initial stages of cancer progression via inducing genomic instability and DNA damage [70] as well as later stages of cancer such as invasion and metastasis [7] by facilitating pathway clearance for invasion of cancer cells caused by ECM degradation [6]. Epithelial-mesenchymal transition (EMT) is also a critical part of tumor invasion and metastasis, where the epithelial cell features are lost in favor of adopting mesenchymal traits, and it involves loss of the apicobasal cell polarity, through intracellular adhesion alteration [71]. MMPs can directly induce the EMT in target epithelial cells which leads to cancer progression [6]. 

The role of specific MMPs and ADAMs in breast [6,72,73], lung [74,75,76,77], prostate [78,79,80], and colorecta [81] cancer progression, invasion, and metastasis has been studied extensively [18,49,82]. MPs carry a multifaceted role in cancer and they can act as a pro-tumorigenic, anti-tumorigenic, or null factor [6], making it difficult to draw a general conclusion on the role of all MPs without considering all the parameters involved in the model of study such as the individual genomic profile, proteomic profile, and experimental conditions. MMP-9, MMP-14, ADAM-12 and ADAM-17 are some of the MPs that were found to have a significant role in several types of cancer.

MMP-9 is the only MMP found in the 70 genes of the Rosetta poor prognosis for breast cancer [83], and is strongly related to the prognosis and poor survival of breast cancer patients [6]. High levels of MMP-9 in serum in breast cancer patients were also correlated with poor outcomes [16]. High expression levels of MMP-9 are also associated with prostate and lung cancer [82]. MMP-9 was shown to be upregulated in the basal-like triple negative and HER2 positive breast cancer cell lines, and MMP-9 knockdown was shown to block metastasis in both triple negative breast cancer cell models and an orthotopic mouse model of human breast cancer [73]. These findings suggest that MMP-9 is a potential therapeutic target for breast cancer as well as other type of cancer and MMP-9-driven diseases. 

MMP-14 is known as one of the key drivers of cancer invasion and progression. MMP-14 was originally found to contribute to cancer progression by activation of proMMP-2 and degradation of ECM to facilitate cancer metastasis. However, MMP-14 activity was later shown to also have a negative effect on immune responses to tumors [84]. MMP-14 also plays a significant role in angiogenesis [84]. Similar to MMP-9, the PEX domain of MMP-14 was shown to be responsible for facilitating cell migration through the molecular cross-talk resulted from the homodimerization of MMP-14 and heterodimerization of MMP-14 with the cell surface adhesion molecule CD44 [85]. Mutagenesis studies in the PEX domain of MMP-14 which is a four-blade β-propeller, showed that blade IV is necessary for MMP-14 homodimerization and that blade I is required for CD44 MMP-14 heterodimerization. The interaction between MMP-14 and CD44 leads to activation of the MAPK and PI3K signaling pathways through phosphorylation of EGFR which will further lead to cell migration [85,86]. 

Other MMPs, such as MMP-1 [87,88], MMP-7 [89], and MMP-11 [90] were also shown to contribute to cancer progression and metastasis [59]. Ovarian cancer cells are found to be leptin-induced and conduct invasion through the MAPK signaling pathway. MMP-7 was found to have a direct role in the activation of the ERK ½ and JNK ½ signaling pathways, which attribute to leptin-induced cell migration and therefore play a pivotal role in these cells [91].

Similar to different MMPs, members of the ADAM and ADAMTS families are known to play a multifaceted role in various aspects of cancer. Many ADAMs are found to play a role in growth factor and cell receptor shedding. For instance, ADAM-12, a multi-domain MP that regulates cell proliferation and movement, is known for shedding of heparin-binding, and EGF-like growth factor (HB-EGFR) to activate the EGFR signal pathway [92]. EGFR, one of the key factors in triple-negative breast cancer (TNBC), is normally activated following the release of ligands, such as TGFα, mediated by ADAM-10 and ADAM-17. ADAM-17, or TACE, is processing membrane-bound TNFa precursor to its soluble form as well as other membrane proteins [93]. A humanized monoclonal antibody targeting both the catalytic domain and the cysteine-rich domain of ADAM-17 was found to significantly inhibit the release of TGFα and decrease downstream EGFR-dependent cell signaling, resulting in reduced proliferation in two-dimensional clonogenic assays as well as growth in a three-dimensional culture of breast cancer cells. Furthermore, the antibody provides a potential anti-cancer therapeutic based on the inhibition of ADAM-17 which resulted in reduced invasion and migration of HCC1143 TNBC mammary gland epithelial cells in vitro [94]. 

ADAM-12 was also found in high levels for both mRNA transcriptional and protein level in tumor tissues, while ADAMTS-1 mRNA and protein levels were significantly lower in non-small-cell lung cancer cells, suggesting distant roles of different metzincin family members [95]. ADAM-12 was also shown to be highly expressed in breast [92], liver [96], and lung cancer [92]. This might be due to ADAM-12’s role in several cell signaling functions such as receptor shedding and binding to activate the Notch signal pathway, the TGF-β signaling pathway, regulation of cell migration, and promotion of angiogenesis [92]. ADAM-12 is upregulated in human breast cancers and is a predictor of chemoresistance in estrogen receptor (ER)-negative tumors. ADAM-12 is induced during the EMT transition, a feature associated with claudin-low breast tumors, which are enriched in cancer stem cell (CSC) markers [92,97].

TIMPs are also involved in cell signaling through regulation of MPs and MMP-independent activities, thus, contributing to different aspects of cancer [27]. TIMP-1 has been also found to support pro-tumorigenic signaling by activating hypoxia-inducible factor (HIF-1), which in turn results in upregulation of microRNA-210 (miR-210) in lung cancer progression [98]. It has also been suggested that TIMP-1 interacts with CD63 to activate ERK ½ kinase which promotes accumulation of cancer-associated fibroblast (CAF) in prostate and colon cancer [99].

TIMP-1 overexpression was shown to facilitate the EMT of hepatocellular carcinoma (HCC) cells via MMP-independent activities such as modulating apoptosis, mitogenic activity, and cellular proliferation and morphology [100]. TIMP-1 upregulation was also found as a prognostic marker for lung metastasis in HCC, since TIMP transcripts were clearly demonstrated in the metastatic HCC nodules in the lung. Similarly, TIMP-2 levels in serum and tissue were decreased in HCC patients with metastasis compared to those without [101], and patients with high levels of TIMP-2 have were shown higher survival rates [102,103]. On the other hand, TIMP-3 was shown to have a potential role in alleviating invasion of HCC, likely through suppressing tumorigenesis and angiogenesis by interacting with integrin α7 and angiotensin II type 2 receptor [103]. TIMP-3 was also shown to inhibit angiogenesis via blocking the binding of vascular endothelial growth factor (VEGF) to its receptor, vascular endothelial growth factor-2 (VEGFR-2) [104,105]. The C-terminal domain of TIMP-3 was found to be responsible for the MMP-independent angiogenesis inhibitory function of TIMP-3 [104]. This VEGF inhibitory property is unique to TIMP-3, and TIMP-1 and TIMP-2 could not inhibit the binding of VEGF to KDR in intact cells. This inhibition appeared to be specific for VEGF, because signaling by PDGF and bFGF through their receptors was unaffected [106]. TIMP-3 downregulation is related to cancer invasion and metastasis in various cancers; however, higher serum level of TIMP-1 is associated with lower response to therapy in breast cancer patients [107], and TIMP-1 has been used as a biomarker along with MMP-2 and ICAM-1 for pancreatic cancer [108,109]. It needs to be considered that higher levels of TIMP-1 expressed in tumor or stroma might be a result of MMP expression in cancer patients [27]. More importantly, TIMP-1 is structurally distant from other TIMPs, and its MMP-independent roles in cell signaling should not be overlooked. 

## 6. MPs in Central Nervous System and Neurodegenerative Diseases

MPs play key physiological and pathological roles in the central nervous system by regulating signaling pathways during neuroinflammation, blood-brain barrier (BBB) disruption, synaptic dysfunction, or neuronal death [8,110]. Upregulation of some of the MPs activity and an imbalance between metalloproteinases and their inhibitors play a key role in the central nervous system, and might contribute to neurodegenerative diseases (ND) such as Alzheimer’s disease (AD), Parkinson’s disease (PD), Huntington’s disease (HD), and multiple sclerosis (MS).

MMPs and TIMPs were shown to be localized in neurotic senile plaques and neurofibrillary tangles in the postmortem brains of patients with AD [111]. Some MMPs were also shown to induce tau aggregation and the formation of neurofibrillary tangles in vitro. Moreover, MMPs play a role in ND pathogenesis via the disruption of the blood-brain barrier and promotion of neurodegeneration [112]. However, MMPs can degrade both soluble and fibrillar forms of amyloid-beta (Aβ). Amyloids are insoluble and resistant to degradation in organs and tissues. Therefore, the formation of amyloids is undesirable and can potentially lead to amyloid diseases such as AD and PD by decreasing the Aβ clearance and providing poor Aβ transport at the blood-brain barrier (BBB) [113,114]. It has also been shown that Aβ upregulates the expression of MMPs in neuroglial cultures and induces the release of TIMP-1 by brain cells. Inhibition of Aβ-induced MMP activity resulted in an improvement of performance tests in mice. Moreover, simultaneous examination of MMP-9, MMP-2, and TIMP-1 in the cerebrospinal fluid (CSF) contributed to the ability to differentiate between AD and other types of dementia [115]. MMP-9 and its endogenous activator, MMP-3, were shown to contributes to AD pathology are associated with synaptopathic neurodegenerative disorders [9,111,116,117]. MMP-9 and MMP-3 are upregulated in the brain tissue of AD patients [118,119]. Overexpressed MMP-9 has also been found in the cytoplasm of neurons, neurofibrillary tangles, senile plaques, and vascular walls of the hippocampus and cerebral cortex of AD patients, and inhibiting MMP-9 improves Aβ-mediated cognitive impairment and neurotoxicity in mice [112]. MMP-9 also has a pro-aggregatory influence on tau oligomer formation in select brain regions, which may contribute to tau’s potential neurotoxic side effects. Further supporting a pathological role of MMP-9 in AD, pharmacologically inhibiting MMP-9 reversed cognitive decline in AD mice treated with an intracerebral-ventricular injection of a broad-spectrum inhibitor of MMPs and reduced neurodegeneration in β-amyloid-treated cultures [112]. Unlike MMP-9, ADAM-10 was shown to decrease Aβ aggregation and alleviates AD progression [120]. Aβ precursor protein (AβPP) is the origin of Aβ, found in humans in only two areas: the brain and cerebrovascular system or skeletal muscle [121]. The creation of Aβ from AβPP is via the processing of three enzymes: α-, β-, and γ-secretase [121]. ADAM10, an α-secretase, was shown to be responsible for degradation of Aβ and most of the proteolytic activities in the CNS [122,123].

Among all MMPs, MMP-2, -3, -9, and -14 were the major MMPs found in the brain [124], and high levels of MMP-9 and MMP-2 were observed in patients with frontotemporal dementia (second most common form of dementia)—a disorder often causing significant personality and behavior changes [125]. In some cases, MMPs can be beneficial to the CNS via regulating synaptic plasticity or even repairing the CNS in adults. In a transgenic AD mouse model (5×FAD), MMP-9 was proposed to be a promising target in AD treatment, capable of interfering with the development and disease progression of AD [126,127]. However, many studies have demonstrated MMPs’ adverse role in the CNS. Lipoprotein receptor-related protein-1 (LRP1) is a key receptor in clearing Aβ [128]. This receptor was found to closely bind to apolipoprotein E (apoE) ligands via different forms in human such as apoE2, apoE3, and apoE4, among which apoE4 embodies the highest risk of AD [113]. It was also found that in human brain microvascular endothelial cells, Aβ concentration and the concentration of soluble LRP1 increase with increased concentrations of MMP-9, suggesting a potential toxicity of MMP-9 in the BBB [113]. SB-3CT, an MMP-9 inhibitor, was also shown to contribute to significant decreases in the level of Aβ and was responsible for about a 40% decrease in soluble LRP1 [113]. The downregulation of MMP-9 has also been shown as a mechanism to regulate neuroinflammation in AD patients [129]. Another investigation also suggested that MMP activation causes neurotoxicity, especially MMP-10 and MMP-14 in HD, another type of dementia [130]. MMPs’ responsibility in advancing neurodegenerative diseases can also be displayed via retinal degeneration, which was exhibited in an MMP-9 knock out mice model [131]. The result of this examination insinuated that MMP-9′s activity negatively affects retinal cells and optic functions [131].

Mutations in microtubule-associated protein tau (MAPT) result in the alteration of protein structure and the availability of different tau isoforms, which later cause impairments in the microtubule assembly and axonal transport, and eventually lead to frontotemporal dementia [132]. Upregulation of MMP-2 and -9 was found to be correlated with some specific tau mutants such as tau-A152T and MAPT IVS10+16 compared to control, which further highlights the roles of MMP-2 and -9 in dementia [125]. The elevated level of MMP-2 and -9 were also contributed to the neuronal cell death of induced pluripotent stem cell (iPSC) with MAPT mutation [125]. Besides the negative effect of MAPT mutations on CNS, the deposition of Amyloid-β (Aβ) plaques has also indicated its undesirable correlation with the severity of dementia, AD, PD, and even HD [121].

MMPs’ involvement in neuroinflammation diseases and neurovascular disorders and neuroinflammation have also been extensively studied. Multiple sclerosis (MS), one of many neuroinflammation phenomena, is considered a remyelination failure disease. Around axons in normal adults’ CNS, new myelin sheaths are created and generated (remyelination) to ensure axons’ conductive properties and to protect axons; however, myelin may be lost or not reconstructed, causing diseases such as multiple sclerosis [133]. Various studies have established the link between MS and MMP activities i.e., high levels of MMP-1, -2, -3, -7, and -12 have been detected in MS patients [134]. The irregular activities of MMPs, specifically MMP-2 and MMP-9, allow the excessive migration of immune cells into the CNS during MS, which are considered undesirable as MS is considered an autoimmune disease [135]. Remarkably, under conditions where the immune system is fragile or compromised, MS is a result of progressive multifocal encephalopathy (PML) disease, caused by John Cunningham (JC) virus or JCV [136]. The correlation between high levels of MMP-9 and the JCV reactivation in relapsing-remitting MS patients was another indicator for the role of MMP-9 in progressive MS [135]. Furthermore, the abundance of MMP-7 and MMP-9 in experimental autoimmune encephalomyelitis, such as MS, suggests the key contribution of specific MMPs in neuroinflammation disorder [137].

Several MMPs including MMP-2, -3, -7, -9, -10, and -1 have also been shown to be involved in causing one of the most fatal diseases in the CNS: stroke [138]. The MMP-8 gene in mice was also found to have a correlation with ischemia risk, and the risk seems to be higher, depending on the cooperation and cross-signaling pathways of multiple MMPs [139]. In stroke, MMP-2 and MMP-9 are considered the two most critical enzymes, and therefore, they were among the ones most studied [140]. MMP-2 and MMP-9 were demonstrated having key roles in increasing oxidative stress and brain matrix remodeling [140]. In mice with hyperhomocysteinemia (HHcy), a vascular dysfunction and stroke-like condition, the protein levels of MMP-2 and MMP-9 were significantly higher than that of the control mice [140]. In a trial of acute ischemic stroke, the patients with high levels of TIMP-1 and MMP-9 also held the highest risks of experiencing major disability or death within 3 months [141]. In another study, high level of active MMP-3 was revealed in the rat’s brain tissue after ischemia [142]. MMP-3 was also detected to be able to cleave and degrade brain agrin, an ECM structure supporting the microvasculature and maintaining the integrity of the BBB [142]. Furthermore, MMP-9 was found to play a role in post-stroke depression. In a population of about 600 ischemic stroke patients and in a period of 3 months after the event, approximately 40% of the patient population were classified to have post-stroke depression symptoms, and the MMP-9 level in these patients (658.8 ng/mL on average) were much higher than those identified without post-stroke depression (485.7 ng/mL on average) [143]. Patients’ blood samples were collected after a minimum of 8 h of fasting for MMP-9 measurements; MMP-9 serum was detected and measured using commercially available ELISA kits [143]. It was suggested that MMP-9 was also involved in cerebral damage following stroke events [143].

## 7. MPs in Cardiovascular Diseases

MMPs have been also long associated with atherosclerosis, one of the most common vascular diseases. Atherosclerosis involves the buildup of major plaque-containing components, such as fat and cholesterol, within the arterial walls, eventually causing abnormal blood movement and later progressing to a series of severe health problems such as stroke, hypertension, heart attack, heart failure, and even death [144,145]. Various members of MMP family were identified to play an essential role in cardiovascular diseases. For instance, high activation of MMPs has the capability to distort the structures of arterial plaques and later burst susceptible buildup plaques [11,115,146]. Other cardiovascular diseases that MMPs may impact are aneurysm (the weakening of arterial walls), diabetes, myocardial infarction, hypertension, cardiomyopathies, periodontitis, hyperglycemia, and dyslipidemia [11,115]. This mechanism can be described generally as such: MMP-2, -3, -9, and -14 cause damages to the vascular smooth muscle cells and endothelial cells [147]. This harmful mechanism then causes the release of TGF-β, which will then lead to vascular calcification, arterial stiffening, and anthogenesis [147].

Among the identified MMPs, MMP-1, -2, -3, -9, and -14 attracted more attention regarding their relation to cardiovascular disorders. MMP-1, -2, -3, -9, and -12 are majorly expressed by fibroblasts [148]. MMP-1, -2, -3, -9, and -12 are all secreted via endothelial cells, while MMP-1 was also shown to be secreted by leukocytes [148]. Other cell types that express these aforementioned MMPs include cardiomyocytes (MMP-2, -9, and -14), macrophages (MMP-2, -3, -9, and -14), vascular smooth muscle cells (MMP-2 and -12), and neutrophils (MMP-9) [148]. Various studies have shown that the increase in the level of active MMP-2 and MMP-9 degrades the ECM and stimulates the rupture of vulnerable arterial plaques [149,150,151]. Other studies have also shown that MMP-1, -3, -9, and -14 contributed to the increase in plaque instability [147]. The correlation between the increase in the expression level of MMP-1, MMP-3, and MMP-12 and plaque instability and cardiovascular disease progression were demonstrated clearly [149]. In the cells infected with influenza a virus, an increase in MMP-13 within 3 days of infection was found [152]. MMP-13 expression, mediated by influenza a virus via p38 mitogen activated protein kinase, was found to destabilize the susceptible arterial plaques triggering cardiovascular events eventually [152]. In patients with carotid stenosis—a narrowing of the carotid arteries, caused by atherosclerosis—MMP-1, -7, and -10 proteins were seen to circulate at a much more significant level/frequency than the controls, providing more evidence and further confirming the hypothesized correlation of MMPs and atherosclerosis [153]. Interestingly, MMP-7 recently showed its promising role as a novel biomarker, in patients with severe carotid stenosis, for recurrent cardiovascular events [153]. The elevated protein expression of MMP-1 and -7 was selected as potential candidates for early death prediction in patients with heart failure [154].

Furthermore, the effects of the MMP family specifically on patients with type 2 diabetes mellitus (T2DM) and advancement of cardiovascular, organ, and tissue damage were shown to correlate with a high level of MMP-12 in T2DM patients [155]. The observation of increased MMP activity in T2DM patients was also made in other studies [156]. As an example, uncontrolled glycemic conditions in T2DM patients were particularly identified with modifications in periodontal tissues; therefore, the severity in periodontitis is analogous to a measurement of T2DM disease progression [157]. Patients with severe periodontitis indicated a considerably higher level of proMMP-2 and MMP-2 than healthy individuals [157]. T2DM is also considered a risk for periodontitis as T2DM elevates inflammation in periodontal tissues in the presence of high levels of inflammatory mediators, i.e., IL-1β, TNFα, nitrites, and MMP activities [153]. Patients with severe periodontitis indicated a considerably higher level of salivary proMMP-2 and MMP-2 than healthy individuals [157]. This result further reinforces the cause-and-effect relationship between high MMP expression and periodontitis and/or T2DM disease progression.

## 8. MPs in Fibrosis and Other Diseases

Epithelial and/or endothelial cells produce MPs in response to an injury, which leads to the activation of cytokines and other growth factors, such as TGF-β, IL-13, and PDGF [158,159]. The dysregulation and overexpression of MPs, cytokines, and aforementioned growth factors cause permanent fibrotic scars [158]. Fibrosis is a condition where various tissues become damaged, thickened, and scarred, mainly due to the excess accumulation of components in the ECM [158]. Fibrosis, which can take place in the lung, liver, or kidney, is a chronic inflammatory response to a number of stimuli such as persistent infections, allergic reactions, chemical and radiation, and tissue injury [158]. MPs play a complex, dual role in fibrosis similar to other diseases. MPs can facilitate fibrosis clearance with proteolytic cleavage of ECM components which works in a favorable direction, however, under special circumstances, upregulation of specific MPs were also found to have an adverse effect which leads to progression of the fibrosis in liver, lung, and kidney. 

Idiopathic pulmonary fibrosis (IPF) has notable consequences, such as excess deposition of components in the ECM, specifically collagen [13]. MMPs were found to be upregulated in pulmonary fibrosis, along with inflammatory agents such as TGF-β and INF-γ, which were also considered as critical fibrosis-inducing factors [160,161]. Inhibition of bleomycin-induced IPF was investigated by bone marrow-derived mesenchymal stem cells. The elevated expression levels of MMP-9, TIMP-1, TGF-β, and INF-γ have been observed in facilitating the IPF disease progression [161]. A significant increase in the levels of MMP-1, ADAM-9, -10, and -17 expression were found by immunohistochemistry that suggested the significant correlation of the overexpression of specific members of MPs in fibrotic lung diseases [162,163]. Various other MMPs were also associated with the pathogenesis of IPF, indicated by the high levels of MMPs in pulmonary cells, including MMP-3, -7, -8, -9, -12, -13, -19, and -28. Some other studies also suggested that the upregulation of MMP-1, MMP-2, -10, -11, and -14 were responsible for the fibrosis progression [13,164]. The highly expressed MMP-2—a producer of structural tissue such as fibroblasts and endothelial cells, and MMP-9—responsible for inflammation in cells related to collagen deposition—were shown to lead to IPF or other fibrosis-related diseases in the lung i.e., Hermansky-Pudlak syndrome [165,166]. In addition, MMP-10 was found to contribute to disease progression of emphysema or shortness of breath due to the impaired lung [167]. Although most studies have indicated the adverse effects of MMPs on IPF, some MMPs could be beneficial in halting disease progression. For instance, MMP-19 was shown to have adverse effects on fibrosis depending on the signaling pathway in some studies [12,163,168], and mice lacking MMP-19 showed more severe symptoms of IPF suggesting MMP-19 could act as a major regulator in pulmonary fibrosis [169]. 

In hepatic fibrosis, TGF-β is probably the most prominent effector. Another effector, such as insulin-like growth factor binding protein related protein 1 (IGFBPrP1), activated hepatic stellate cells (HSC). These cells then produced α-smooth muscle actin (α-SMA), which contributed greatly to the ECM secretion and deposition [170], thus, suggesting that IGFBPrP1 regulated the balance between MMP/TIMP level in liver fibrosis models. It was also found that the level of UGFBPrP1, along with α-SMA and TGF-β1, increased during fibrogenesis [170]. Moreover, more studies showed that MMP-14, MMP-2, -3, -8, -10, -12, and -13 among other MMPs were significantly elevated during liver injury, resultantly speeding up the process of fibrosis formation [171,172]. Mounting evidence has exhibited that MMP-9 is able to activate TGF-β, accelerating the fibrogenesis process [173]. Interestingly, MMP-9 is also recognized to have the ability to both generate and resolve fibrosis in the liver [173]. MMP-9 resolves fibrosis via decreasing collagen I and assisting in the programming of the activated HSC cell death pathway [173]. However, more study still needs to be done to understand the indirect fibrosis resolution properties of MMP-9.

The MMP family also plays a role in chronic kidney disease, a consequence of diabetic kidney disease (DKD). Similar to the fibrosis development in the lung, heart or liver, the excess deposition of components in the ECM causes the thickening of the basement membranes. This eventually leads to sclerosis and tubulointerstitial fibrosis, the hallmark of DKD pathogenesis [174]. Besides the major players, MMP-2 and MMP-9, TGF-β1 is recognized as a key factor that takes parts in the nephrotic fibrosis [175]. Considering children with nephrotic syndrome displayed a higher level of MMP-1, -2, and -9, these MMPs have the potential to act as biomarkers for this disease [176]. However, one recent study has presented that the diabetic children urinary fibrosis biomarkers (levels of MMP-2, MMP-9, and TGF-β) versus the control group showed no significant difference [175]. Therefore, the role of MMPs in DKD is not yet fully understood. There is also evidence that other MMPs play a role in kidney fibrosis. For example, MMP-10 upregulation is responsible in early stage of DKD [177]. MMP-7 was also found to contribute to kidney fibrosis via 3 pathways: EMT, TGF-β, and ECM deposition [173]. The MMP-7 expression level was also evaluated in each pathway, which was found out to be strongly associated with fibrosis development and complications in renal diseases [178].

**Figure 2:**
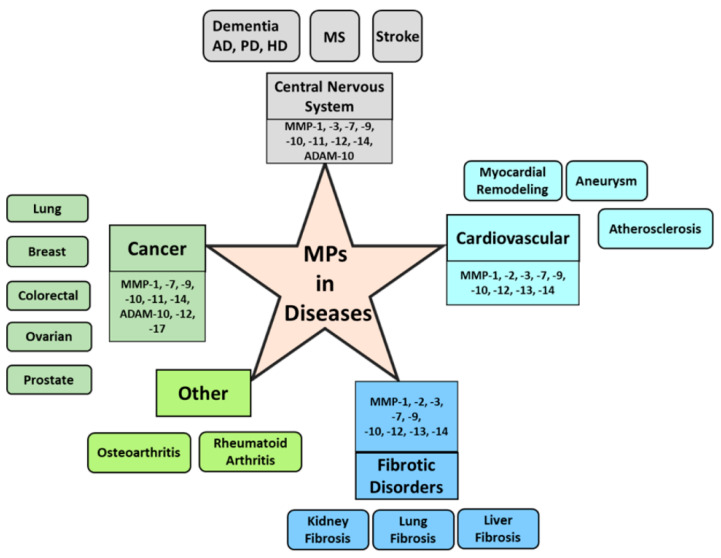
Metalloproteainses (MPs) in developing diseases.

MMP, ADAM, and ADAMTS also play a critical role in other diseases, such as rheumatoid arthritis and osteoarthritis, by the degradation and remodeling of extracellular matrix proteins and the shedding of cell signaling receptor molecules. Although MPs are responsible for the maintenance of homeostasis in normal conditions, the pro-inflammatory cytokines may affect the upregulation of MMP, ADAM, and ADAMTS production in inflamed synovial joints [179], which leads to joint damage and progression of rheumatoid arthritis and osteoarthritis. A summary of MPs responsible for developing several diseases is depicted in Figure 2.

## 9. MMP Inhibition for Developing Therapeutics

Upregulation of specific MMPs have been long associated with several diseases, and downregulation and inhibition of specific MMPs were proven to be effective in alleviating several diseases. Several MMP knockdown studies in mice were conducted to prove this point [73,88,180,181,182,183]. Thus, inhibition of MMP catalytic activity provides a conventional strategy to inhibit progression of these diseases, and MMP inhibitors may offer potential therapy. However, MMP inhibitors based on small molecules such as marimastat and rebimastat previously failed in late stages of the clinical trials due to lack of selectivity in targeting the catalytic domain of proteases. Most of these small molecules were zinc ion chelators and not specific to any MMP. Therefore, there is still a lack of efficient MMP inhibitors that selectively target pathological MMPs [184]. Because of the extensive role of MPs in cell signaling and immune system, inhibition of MPs may also enhance immunotherapy since it was shown that inhibition of MMP-9 and MMP-14 improved T-cell anti-tumor response [185,186].

Several members of the MMP family have overlapping substrate specificity, which makes it challenging to narrow down the specific function of each MMP and their role in ECM. Protein-based MMP inhibitors provide more selective therapeutics. TIMPs, as natural inhibitors, and antibodies are two main groups of protein antagonist against MMP targets. TIMPs bind to the MMP catalytic site with their N-terminal domain, known as the inhibitory domain. TIMPs neutralize activity of MMPs, which is a key feature in using them as potential therapeutics.

### 9.1. Small Molecules

The first generation of MMP inhibitors were based on small molecules and relied on targeting the zinc ion in the active site of MMPs. This class of compounds contain a hydroxamic acid motif, which makes a potent interaction with the catalytic zinc ion of MMPs [187]. The use of hydroxamate-containing compounds rapidly grew since these compounds were in clinical use and several patents already existed for them. However, partial instability and hydrolysis of hydroxamate to hydroxylamine and carboxylate derivates, along with lack of selectivity between several MMPs, ADAMs, and other zinc proteases resulted in failure in later stages of clinical trials for this class of MMP inhibitors [49,69]. Designing efficient molecular inhibitors requires precise understanding of the structural details of MMP proteins. Another class of MMP inhibitors were produced to fit the variable S1′ pocket of MMPs [15,188,189,190,191]. MMPs contain a S1’ pocket located in close proximity to the catalytic zinc (II) ion which is important for the recognition of the preferred substrate [192]. Structural insights such as understanding the impact of modifying the S1’ pocket of MMP-12 on the interactions between inhibitor drug molecules and MMPs, facilitated the design of efficient MMP inhibitors [192]. MMP-13, among other MMPs, is forming a unique side pocket (S1’*) after ligand binding to a large S1’ pocket. Novel MMP-13 inhibitor molecules have been designed based on pyrimidine derivatives interacting with catalytic zinc in the S1’ pocket [193], or with the aromatic residue in the S1’* pocket instead of the catalytic site with zinc ion [194]. 

MMP inhibitors were also designed to target the enzyme exosites. A set of branched amphiphilic polymers were developed based on understanding the allosteric mechanism and dynamic interaction between two domains of MMPs [195,196]. These polymers were used for the recognition of hidden allosteric regulatory sites on the surface of MMP-12 and MMP-14- catalytic domains [197]. The inhibition mechanism for this class of inhibitors is based on polymers that compromise catalytic activity by interacting with flexible surfaces of MMP-12 and -14 that are known to have a high mobility. The allosteric regulatory sites found in this study were similar to predictions from computational analyses of 13 MMP family members [197]. Computational studies were also used to find small molecule inhibitors that target PEX domains of MMPs. In one study, a small molecule inhibitor was identified that blocked MMP-9 [198] and MMP-14 [199] dimerization selectively by targeting the PEX domain without affecting the catalytic activity. The selective MMP-9 inhibition resulted in inhibition of cellular migration in a mammary tumor model and mammary tumor growth and metastasis in an orthotopic xenograft. Another highly selective MMP-9 compound was identified using a novel mechanism which blocks MMP-9 activation. This small molecule, known as JNJ0966, was discovered through a biochemical and structural screening. JNJ0966 was shown to bind to the structural pocket in proximity to the MMP-9 zymogen cleavage site near the Arg106 residue. It was likely that JNJ0966 inhibition was through an allosteric perturbation that reduced cleavage of pro-MMP-9 at the Glu-59/Met-60 site [200]. The compound showed no inhibition toward the catalytic activity of MMP-1, MMP-2, MMP-3, MMP-9, or MMP-14, and activation of pro-MMP-2, an MMP closely related to MMP-9, further proving its high selectivity in binding and inhibition of pro-MMP-9 [200]. JNJ0966 was applied in the neuroinflammatory mouse EAE model orally which significantly resulted in eliminating clinical disease score, showing the therapeutic potential of inhibiting proMMP-9 activation as a therapeutic approach [200].

### 9.2. Peptides

Collagens consist of three α chains with Gly-X-Y repeats (where X and Y are often Pro and Hyp). Triple Helical Peptides (THP) that mimic the triple helical collagen substrate of MMPs were developed as efficient inhibitors that target various domains of MMPs [201]. Collagenase MMPs such as MMP-13 recognize collagen I, II, and III with substrate preference of collagen II over the other two. In a recent study, a library of overlapping homotrimeric peptides forming THP were tested for MMP-13 binding. The triple-helical conformation of the peptide as well as the PEX domain of MMP-13 were determined to be necessary for efficient binding of peptide toolkits to MMP-13 [202]. THPs with a (Gly-Pro-Hyp) 5 sequence repeat and phosphinate transition state analogues were used to target the catalytic domain and the exosites on the fibronectin domains of MMP-2 and MMP-9 with high affinity and selectivity. These THPs were designed for selective inhibition of the proteolytic activity of MMPs that blocks cleavage of type V collagen but not interstitial collagen [203]. More recently, heterotrimeric THPs were also synthesized using click chemistry as selective binders to MMP-13 and MMP-14, with a high selectivity between MMP-1 and MMP-14 [204]. 

### 9.3. Protein-Based MP Inhibitors

Although small molecule drugs can be well-designed, especially when the crystal structures of MMPs are available [205,206], they carry a broad-spectrum binding to MMP/ADAM/ADAMTS family, and therefore, suffer from low selectivity and high toxicity. On the other hand, MMP inhibitors based on protein therapeutics such as antibodies and TIMPs offer a superior potential for higher selectivity due to the large antigen–antibody, or TIMP-MMP protein–protein interaction surface, and therefore, reduced toxicity. Furthermore, protein engineering techniques such as directed evolution and surface display [207] can be used to improve the affinity, selectivity, and stability of this class of MMP inhibitor drugs. 

#### 9.3.1. Antibodies

Antibodies have been extensively developed and engineered as therapeutic agents [207,208,209]. Antibody therapeutics usually have a well-defined action mechanism and both low immunogenicity and toxicity [192]. Several antibodies were found to selectively block MMP-9 activity within a lower nanomolar range affinity by targeting the surface of the catalytic domain [210,211].

Rational design of antibodies based on computational modeling and site-directed mutagenesis of an antibody resulted in discovering an MMP-14 inhibitor that binds to the catalytic domain, blocking the interaction between MMP-14 and TIMP-2, and therefore, disrupting the proMMP-2 activation [212]. Phage display was used to develop a selective monoclonal antibody inhibiting MMP-14. This antibody was able to inhibit tumor growth and metastasis in an orthotopic xenograft model of breast cancer [213], or inhibit lymphangiogenesis, while other catalytic functions were unaffected [214]. MMP-blocking antibodies were also generated in mice immunized with a synthetic MMP active site. The antibodies, produced using this method, were able to bind to MMP-2 and MMP-9 with a mechanism similar to natural MMP inhibition [215], and also to two exposed loops of MMP-14 which resulted in allosteric inhibition of catalytic function [216]. 

Several monoclonal antibodies (mAb) were developed targeting MMP-9 [6,81,217]. Five mouse mAbs were isolated using sandwich ELISA and hybridoma technology from a mouse serum prepared using MMP-9 as an antigen. Among those, two showed strong binding affinity to MMP-9 while one of them, REGA-3G12 with nM binding affinity toward the MMP-9 catalytic domain, acted as a selective inhibitor of MMP-9 [210]. A single chain variable fragment (scFv) of REGA-3G12 was also found as a selective MMP- 9 inhibitor compared to MMP-2, demonstrating a highly potent selective MMP-9 inhibitor capable of distinguishing between two closely related MMPs (MMP-2 and MMP-9) [218]. Interestingly, an oligohistidine tag incorporated in one of the mAb derivatives was also shown to have MMP-inhibitory effects, most likely due to interactions with the catalytic zinc ion [218]. Two other mAbs targeting MMP-9, AB0041 and AB0046, were found to inhibit tumor growth and metastasis in a surgical orthotopic xenograft model of colorectal carcinoma [81] while reducing the musculoskeletal syndrome usually caused as a side effect of non-selective MMP inhibitors [81]. Andecaliximab is a humanized mAb of AB0041, and was found to have a dual inhibitory function of both active MMP-9 and pro-MMP-9 since it interacts with a region located between the catalytic and pro-domain of MMP-9. However, it has a weaker binding affinity to MMP-9 compared to pro-MMP-9 [219]. Andecaliximab derivatives were evaluated in clinical trials for ulcerative colitis proven to be safe in early stages of clinical trials [220]. AB0046 was also shown to have improved immune responses to tumors, as the inhibition of MMP-9 reversed MMP-9 inactivation of T-cell chemoattractant CXCR3 ligands (CXCL9, CXCL10, and CXCL11) [185]. Safety and pharmacokinetics of Andecaliximab were evaluated in a phase 1b trial for rheumatoid arthritis patients (ClinicalTrials.gov Identifier NCT02176876), proving the selective anti-MMP-9 mAb safe for clinical use with low-severity adverse events and none of the undesired off-target effects that normally cause the musculoskeletal syndrome [221]. Table 1 includes the representatives of each class of MMP inhibitors in clinical trials.

Protein engineering approaches such as directed evolution and high throughput screening were applied to engineer antibodies with higher affinity and selectivity toward specific MMPs. A convex paratope of the Fab domain of antibody library was panned using phage display, and analyzed in-depth using next-generation sequencing. A group of highly potent and highly selective Fabs inhibiting the catalytic domain of MMP-14 were identified with nM range affinity which were not isolated by ELISA [231]. Cyclic MMP-14 inhibitory peptides with or without terminal cysteines (CFSIAHEC or FSIAHE) were utilized as the inhibition warhead. The inhibitory peptide called peptide G was inserted into one of the complementary determining regions (CDR) in the heavy chain (CDR-H3) of the human antibody Fab library. A selective Fab binder was isolated using phage panning with more than 1000-fold enhancement for MMP-14 inhibition. The Fab mutant with peptide G grafted into the CDR-H3 region was also able to selectively bind to MMP-14 in the presence of the MMP-14 natural inhibitor (n-TIMP-2) [232], demonstrating its potent inhibitory function. 

#### 9.3.2. TIMPs

Human TIMPs are natural inhibitors of MPs, blocking MMP, ADAM, and ADAMTS catalytic activity by binding to the MP catalytic site with a wide range of affinity [16,233]. Four types of TIMPs in humans, with about 21 kDa protein size sharing less than 40% amino acid sequence homology [27], were studied extensively for their role in MMP and ADAM inhibition and therefore, offer strong potential protein scaffolds for targeting specific MMPs [27,32,234].

Although TIMPs play a key role in MMP inhibition, they also interact with various cell signaling molecules mediating cell differentiation and proliferation [27,37,235]. The MMP-independent protein-protein interactions of TIMPs make them controversial protein scaffolds for prognosis and treatment of diseases as discussed in the ‘MPs in cancer’ section [27]. For instance, TIMP-1 is known as an MMP-9 inhibitor, and overexpression of TIMP-1 is expected to show anti-tumorigenic properties. Earlier studies showed controversial results for correlation between TIMP-1 expression and tumorigenesis, and elevated levels of TIMP-1 expression resulted in an increase in tumor growth [236,237], or reduced cell invasion in other cases [238,239]. However, more recent investigations demonstrate that high levels of TIMP-1 are related to poor prognosis in some clinical tests [27,184]. In a recent study, this adverse relation has been linked to TIMP-1 inhibition of tumor cell death by activating phosphoinositide 3-kinases (PI3Ks), which are responsible for AKT/ERK phosphorylation [98]. Thus, understanding details of cell signaling functions of TIMPs in progression of diseases is critical to develop efficient therapeutics based on TIMPs. 

TIMPs bind to a broad range of MPs with a range of affinity from pM to nM. Thus, developing TIMP scaffolds that target specific MPs with high selectivity requires engineering TIMP binders. Mutagenesis studies have found mutations responsible for enhancement in selectivity of TIMPs towards MMPs [240,241,242,243,244,245,246]. Computational and experimental analysis of TIMP-2 has revealed specific mutations that improve its affinity for binding to MMP [247]. The affinity of TIMP-1 toward MMP-14 was improved by more than an order of magnitude by replacing a single amino acid in the binding interface [243]. This involved reducing the flexibility of the interacting loop by decreasing the entropy of TIMP/MMP complex formation [248]. In the effort of developing strategies to fine-tune TIMP selectivity toward specific targets, a mutant TIMP was designed for selective MMP-14 inhibition, which blocked collagenase activity and CD44 shedding in cell culture models of breast cancer and fibrosarcoma [249]. Rational design of TIMP scaffolds requires a detailed understanding of TIMP/MP structure and mechanism, which might not be feasible for every pair of TIMP/MP where the crystal structure is not available. Even with a high-resolution crystal structure, predicting the impact of mutagenesis of each single residue is out of reach.

**Figure 3:**
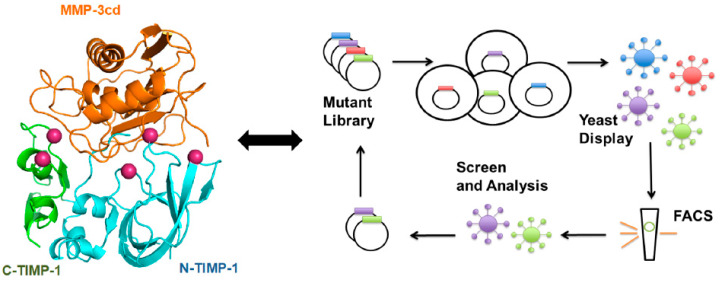
Engineering and design of tissue inhibitors of metalloproteinases (TIMP) scaffolds for targeting specific MMPs with high affinity and selectivity. Combination of rational design (left) and directed evolution using yeast cell surface display and high throughput screening (right) were used to engineer TIMP-1 variants targeting MMP-3 [32]. TIMP-1mutant; shown in blue (N-terminal domain) and green (C-terminal domain), in complex with MMP-3 catalytic domain (MMP-3cd); shown in dark orange protein crystal structure (PDB ID: 6N9D) was shown with the potential regions for random mutation highlighted with magenta spheres.

The structural studies combined with directed evolution of the N-terminal domain of TIMP-2 (N-TIMP-2) was used to develop selective MMP-14 binders. The N-TIMP-2 mutants displayed on the yeast surface were screened using Fluorescent-Activated Cell Sorting (FACS) for high affinity and selectivity towards MMP-14, resulting in more than a 500-fold increase of selective inhibition of MMP-14 [250]. Directed evolution using yeast surface display provides a robust platform for high throughput screening [207] of TIMP scaffolds without the need of detailed knowledge of the protein structure or computational studies for each pair of TIMP/MP complex. However, directed evolution is also limited by the size and quality (e.g., frequency and combination of mutations) of the library of variants. To bring the best of the two worlds together and increase the probability of hitting the TIMP variants with the highest affinity and/or selectivity toward MMPs, a targeted library of TIMP-1 mutants focused on the residues at the interface of TIMP-1/MMP-3 was generated and screened toward MMP-3 binding [32] (Figure 3). 

The isolated TIMP-1 mutants showed an order of magnitude improvement in binding to MMP-3 catalytic domain (MMP-3cd). Although most of the focus for engineering TIMPs were focused on the N-terminal/inhibitory domain of TIMPs [103,251,252], it was shown that cooperation between N- and C-terminal domains of TIMP-1 improved binding to the target MMP-3.

Unlike antibodies, which do not always target the MMP catalytic site, TIMPs naturally neutraliz MMP inhibitors with high affinity, evolved to regulate metalloproteinase activity. The TIMP interaction surface with MMPs contains six main regions (CTC motif at the N-terminus, AB-loop, C-connector loop, GH-loop, EF-loop, and MTL-loop), which resemble monoclonal antibody (mAb) IgG interacting loops located in the light and heavy chains [6]. Several mAb therapeutics are approved by the FDA and are in the market as efficient drugs against several diseases such as cancer and rheumatoid arthritis. Thus, similar techniques for isolation, large-scale production, purification, and engineering of TIMP-based scaffolds can be adopted from mAb production pipeline. Considering MMP-independent activities of TIMPs [37,253,254], it is important to define the sequence and structural properties of TIMPs using structural and mutagenesis studies to design highly selective designer protein scaffolds targeting pathological MPs. Understanding MMP-independent function of TIMPs is also critical to engineer and design next generation of TIMP scaffolds as efficient therapeutics (Figure 3).

## 10. MMP-Activated Drugs and Drug Delivery Tools

**Figure 4:**
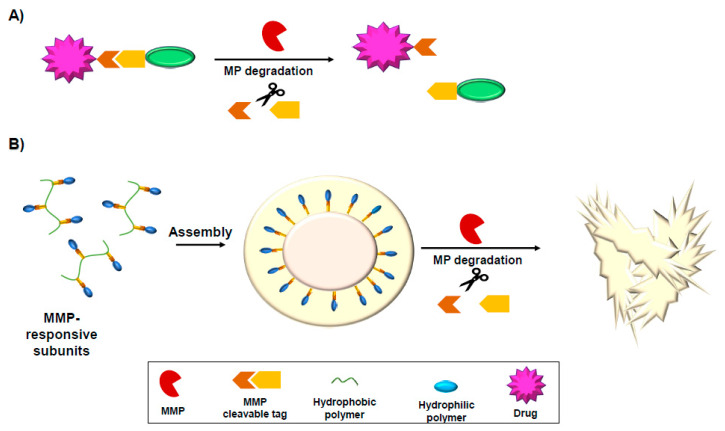
MMP-responsive therapeutics—(**A**) Activation of pro-drug by MMP degradation; MMP cleavable tag (yellow/orange arrow) is attached to the inactivated drug. The drug gets activated by cleavage of the MMP substrate, (**B**) MMP-resonsive nanoparticles, containing hydrophilic–hydrophobic elements, go through transition rearrangement after exposure to the MMP degradation.

One of the major concerns in drug delivery and reducing toxicity of drugs is to improve site-specific drug delivery. Several approaches were used for efficient delivery of therapeutics based on overexpression of MMPs at the tumor site. These drug delivery approaches are based on MMP-activated prodrug or MMP-degradable drug carriers such as nanoparticles or hydrogels (Figure 4). 

### 10.1. MMP-Responsive Prodrugs

MMP-activated prodrugs contain an MMP degradable sequence linked to a cytotoxic motif which controls the activity and cytotoxicity of the agent. Upon cleavage of the MMP sequence, the prodrug gets activated (Figure 4A). One example of an MMP-activated therapeutic is an anthrax toxin protective antigen (PA) that was fused to an MMP cleavage site. PA got activated by MMPs overexpressed at the tumor cell surface which led to improved cell death at the tumor site due to the site-specific delivery of the active drug [255]. The recombinant cytotoxin, FP59, consisting of anthrax toxin lethal factor residues 1–254 fused to the ADP-ribosylation domain of Pseudomonas exotoxin A, was internalized upon MMP-dependent activation of PA. MMP inhibition via small molecule or TIMP-2 resulted in blocking the toxicity of PA. MMP overexpression in tumor cells was required for recombinant PA proteins to kill the cells. It was found that only tumor cells were destructed in a coculture model while the normal cells survived, demonstrating the great potential of site-specific delivery to tumor sites [255]. 

There are challenges in using MMP-activated prodrug approaches for site-specific drug delivery. MMP proteolysis usually leaves some residues behind after MMP cleavage. This remaining residues and chemical conjugation of drugs to MMP-cleavable peptides might alter cytotoxicity or proper targeting of the drug [256]. 

### 10.2. MMP-Responsive Hydrogels

Polyethylene glycol (PEG) hydrogels are extensively used as drug delivery vehicles because of their biocompatibility, hydrophilic tendency and tunable degradation profiles [257,258]. Developing an MMP-responsive hydrogel with a fine-tuned control-release mechanism makes this a strong platform for smart delivery of therapeutics. An MMP-8-degradable hydrogel was synthesized from diacrylate-containing polyethylene glycol–based scaffolds and a cysteine-terminated peptide crosslinker (CGPQG↓IWGQC) which is the MMP recognition tag [259]. The peptide GPQGIWGQ, derived from a type I collagen and a substrate for several members of the MMP family, was used to form a hydrogel by cross-linking vinyl sulfone-functionalized multi-arm PEG for tissue remodeling. The thiolate groups of bis-cysteine peptides were conjugated in a reaction that is feasibly close to physiological conditions (e.g., temperature and pH) [257]. Digital time-lapse microscopy and image analysis were used to demonstrate the MMP-sensitive hydrogel response to MMP regulation using analysis and quantifying cell migration [257]. Other MMP-responsive PEG hydrogel microparticles were synthesized using an emulsion polymerization. A GPQGIFGQK peptide sequence, known as MMP-1 substrate, was incorporated in the hydrogel microparticle. These hydrogel microparticles were shown to be nontoxic and their viability as drug carriers was demonstrated by an MMP dose-dependent controlled release for three types of drugs: a hydrophobic compound (dexamethasone), a hydrophilic compound (methylene blue) and a protein (horseradish peroxidase) [260]. Another MMP-cleavable peptide, acetyl-Cys-Gly--Leu-Asp-Asp was incorporated into a poly (acrylic acid-co-methyl methacrylate) hydrogel with cisplatin (a chemotherapeutic agent), which did not show toxicity in NIH/3T3 fibroblast cells [261], proving its potential for drug delivery. The hydrogel microparticles responsive to MMP-2 and MMP-9 degradation were further used for delivery to malignant glioma cell lines shown to enhance the release rate and reduce cytotoxicity [14].

An MMP-8-responsive drug delivery hydrogel was also formed with quadridentate PEG-DA as the backbone and MMP-8-degradable peptides GPQG↓IWGQ harboring two cysteines at two termini as the crosslinkers. Bovine serum albumin (BSA) and minocycline hydrochloride (MH) were encapsulated in the hydrogel and delivery using MMP-8, and showed no negative effect on hydrogel physical properties. Enzyme-sensitive hydrogels require distinctive pore size to enable enzymes to diffuse into the hydrogels, therefore making it important to design the MMP-responsive hydrogels based on the MMP and drug size [259].

### 10.3. MMP-Responsive Nanoparticles

Nanoparticles were also developed with triggered disassembly or morphology change to improve site-specific delivery of drugs to the tumor site [262,263]. A gelatin-albumin (Gel-Alb) hybrid nanoparticle was generated and evaluated for its drug efficacy as an anti-cancer drug delivery system in a panel of subtype-specific breast cancer cell lines. A selective tropomyosin receptor kinase A (TrkA) inhibitor was trapped inside the Gel-Alb nanoparticle and its anticancer effects in breast cancer were demonstrated [264]. MMP-responsive nanoparticle micelles that contain hydrophobic/ Hydrophilic tails and adjust their morphology can adapt their morphology and size upon exposure to MMP proteases and disassemble from nanoparticle micelles to micrometer aggregates [265], or nanotubes [266]. The self-assembled nanoparticle of drug conjugated hyaluronic acid (HA)-tagged with MMP-cleavable peptides formed a nanofiber with a diameter of 30–40 nm and length of 200–300 nm, which was shown significant improvement in efficient targeting of drug resistant cancer cells [266] (Figure 4B). 

Another MMP-degradable nanoparticle was developed based on a recombinant Sendai virus (SeV). An MMP-cleavable sequence was inserted within the SeV. The recombinant SeV was shown to destruct the human tumor cells expressing matrix metalloproteinases in a selective manner [267]. The recombinant SeV was lacking the matrix protein which prevented effective virus assembly. An MMP-9 substrate sequence (Pro-Leu-Gly---Met-Thr-Ser) was incorporated to the viral fusion cleavage site and altered to render it susceptible to MMPs. This recombinant SeV was able to spread only in the cancer cell lines where MMP was present. As a result, the tumor cell progression in HT1080 human fibrosarcoma mouse models was reduced [267]. A human pancreatic epithelioid carcinoma cell line, Panc I, with lower MMP-2 and MMP-9 expression levels than HT1080 cells was unable to activate the recombinant SeV [267], demonstrating this system as an efficient tool for delivery to tumor sites where MMP-2 and MMP-9 are overexpressed.

Drug delivery techniques have progressed from passive tumor targeting to active targeting of cancer associated receptors and ligands. MPs have great potential for developing stimuli-responsive pro-drugs, nanoparticles, and hydrogels that get triggered in the tumor site, yet remain intact and harmless in healthy cells. Fine-tuning these smart therapeutics and delivery vehicles that selectively respond to specific MPs will bring the next generation of efficient drug delivery tools.

## 11. Discussion and Future Directions

Metalloproteinases (MPs) play a significant yet complex role in several diseases, including cancer, neurodegenerative, cardiovascular, and fibrotic diseases, through several proteolytic and cell signaling functions that they carry within the ECM. MPs are responsible for degradation of ECM, stromal connective tissue, and BBB tight junctions, and thus, are the driving factors in cancer invasion and metastasis, progression of neurodegenerative diseases, and other pathological disorders. MPs and their inhibitors, TIMPs, also regulate cell signaling functions via interacting with growth factors and cell receptors that are important in several diseases.

Fine-tuning regulation of specific MPs responsible for pathological disorders is a key to developing highly efficient therapeutics. Although the cellular function of MMPs, ADAMs, ADAMTSs, and TIMPs were extensively studied, a “higher-resolution picture” of how these MPs and their inhibitors are orchestrated along with other cell receptors within the ECM is required in order to fully harness the potential of the MP inhibitors as efficient therapeutics. Directed evolution of protein-based MP inhibitors that target specific MPs with high selectivity using high throughput screening techniques provides deep exploration of the vast mutational space of these variants. However, to increase the probability of isolating variants with the highest selectivity, deep understanding of the structure and function of MPs and their inhibitors are required. Furthermore, the full potential of therapeutics targeting specific MPs will be harnessed by developing ‘smart,’ MP-responsive drug delivery vehicles.

## Figures and Tables

**Table 1:** MMP inhibitors in clinical studies.

MMP Inhibitor	Type	Clinical Trial	Specificity	Reference
Periostat (doxycycline hydraxate)	Small molecule	FDA approved	Broad spectrum	[222,223]
IImostat; GM6001(Hydroxamate derivative)	Small molecule	Phase I, II	MMP-1, -2, -9	[224]
Marimastat (BB-2516)	Small molecule	Phase II	Broad spectrum	[225]
Prinomastat (AG-3340)	Small molecule	Phase III	MMP-1, -2, -9	[226]
Batimastat (BB-94)	peptide	Phase I	Broad spectrum	[227,228]
GA-5745/andecaliximab	mAb	Phase I, II, III	MMP-9	[221,229,230]
